# Investigation of hydrophobic moment and hydrophobicity properties for transmembrane *α*-helices

**DOI:** 10.1186/1742-4682-1-5

**Published:** 2004-08-16

**Authors:** James Wallace, Onkabetse A Daman, Frederick Harris, David A Phoenix

**Affiliations:** 1 Department of Physics, Astronomy and Mathematics, University of Central Lancashire, Preston, PR1 2HE, UK; 2 Department of Forensic and Investigative Science, University of Central Lancashire, Preston, PR1 2HE, UK; 3 The Dean's Office, Faculty of Science; University of Central Lancashire, Preston, PR1 2HE, UK

**Keywords:** Hydrophobic moment, window size, angular frequency, transmembrane protein, *α*-helix

## Abstract

Integral membrane proteins are the primary targets of novel drugs but are largely without solved structures. As a consequence, hydrophobic moment plot methodology is often used to identify putative transmembrane *α*-helices of integral membrane proteins, based on their local maximum mean hydrophobic moment (<*μH*>) and the corresponding mean hydrophobicity (<*H*>). To calculate these properties, the methodology identifies an optimal eleven residue window (*L *= 11), assuming an amino acid angular frequency, *θ*, fixed at 100°.

Using a data set of 403 transmembrane *α*-helix forming sequences, the relationship between <*μH*> and <*H*>, and the effect of varying of *L *and / or *θ *on this relationship, was investigated. Confidence intervals for correlations between <*μH*> and <*H*> are established. It is shown, using bootstrapping procedures that the strongest statistically significant correlations exist for small windows where 7 ≤ *L *≤ 16. Monte Carlo analysis suggests that this correlation is dependent upon amino acid residue primary structure, implying biological function and indicating that smaller values of *L *give better characterisation of transmembrane sequences using <*μH*>. However, varying window size can also lead to different regions within a given sequence being identified as the optimal window for structure / function predictions. Furthermore, it is shown that optimal periodicity varies with window size; the optimum, based on <*μH*> over the range of window sizes, (7 ≤ *L *≤ 16), was at *θ *= 102° for the transmembrane *α*-helix data set.

## Background

Integral membrane proteins are the primary choice as targets when developing new drugs and although clearly of medical relevance, forming 20% – 30% of the gene products of most genomes, these proteins have been structurally determined in only about thirty cases [1,2]. Where high levels of sequence homology exist, an unknown protein's structure and hence, the location of its membrane interactive segments, can sometimes be deduced by direct comparison to known protein structures. However, where sequence information alone is available, the identification of transmembrane *α*-helical structure requires a bioinformatics approach to understanding the structure / function relationships of these *α*-helices. A number of *α*-helical properties have been used as models to study transmembrane *α*-helices and their structure / function relationships but the most commonly used are those based on the amphiphilicity of protein *α*-helices with the hydrophobic moment used as a measure of amphiphilicity [3].

To quantify the amphiphilicity of protein secondary structures, Eisenberg and co-workers [4] introduced the hydrophobic moment, *μ*(*θ*), which provides a measure of the structured partitioning of hydrophilic and hydrophobic residues in a regular repeat structure of period *θ*. For a structure comprising *L *consecutive residues, the general form of *μ*(*θ*) is given by:



where *H*_*j *_is the hydrophobicity of the *j*^th ^residue within the sequence, and *θ *is the angular frequency of the amino acid residues forming the structure. Eisenberg *et al*., [4] assumed that for an *α*-helix, *θ *is fixed at 100°, and that a segment of eleven consecutive residues, equivalent to three turns of an *α*-helix, could be used to represent amphiphilic *α*-helices. These assumptions led to the more generally used measure of *α*-helix amphiphilicity, the mean hydrophobic moment <*μH*>, where

<*μH*> = *μ(100°)/11*

**Figure 1:**
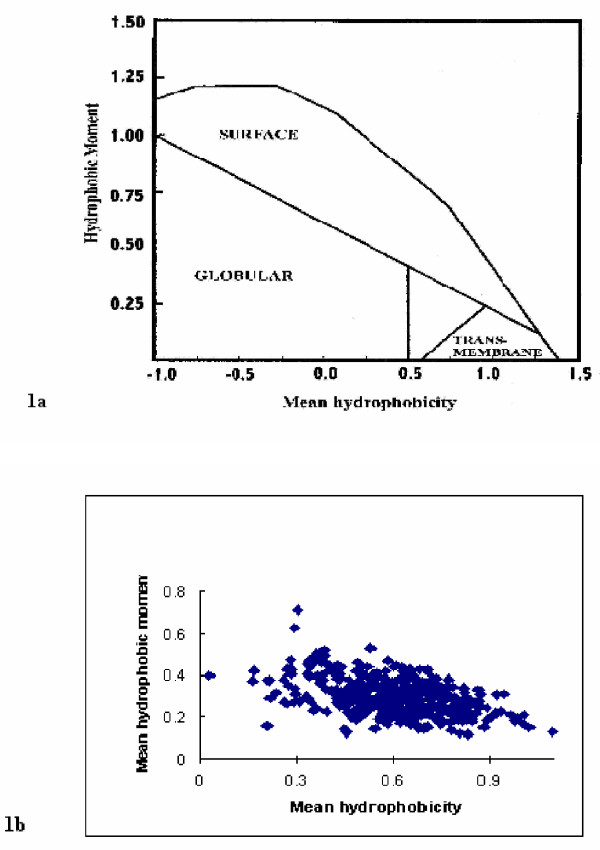
**Conventional hydrophobic moment plot analysis of the transmembrane protein data set. **Figure 1a shows the hydrophobic moment plot diagram [5] with protein classification boundaries. Figure 1b shows the results of hydrophobic moment plot analysis of the 403 transmembrane sequences of our data set using the conventional values of *L *= 11 and *θ *= 100° [4].

As a major extension to the use of the hydrophobic moment, Eisenberg *et al*., [5] introduced hydrophobic moment plot methodology, which provides a graphical technique for the general classification of protein *α*-helices. Using this methodology, a putative protein *α*-helix is characterised according to its maximum <*μH*> and corresponding mean hydrophobicity, <*H*>, where this is defined by:



The parameters <*μH*> and <*H*> are then plotted on the hydrophobic moment plot diagram (figure 1) and the location of the resulting data point used to classify the putative *α*-helix.

The mean hydrophobic moment is widely used and generally regarded as a good predictor of *α*-helix amphiphilicity but the results of statistical analyses have shown the efficacy of hydrophobic moment plot methodology as a predictor of *α*-helical class to be less certain [6]. A number of authors have observed that the methodology can erroneously classify *α*-helices in cases where the hydrophobic moment for a particular amino acid sequence is greatly affected by the spatial arrangement of a few extreme amino acids, thus masking the overall nature of an *α*-helix [3]. However, a more fundamental source of erroneous classification could come from the questionable assumption made by hydrophobic moment methodology with respect to angular periodicity. It is known that in naturally occurring *α*-helices, *θ *can vary over the range (95° ≤ *θ *≤ 105°) and between consecutive residues [7]. Clearly, assuming a fixed value of *θ *= 100° for all *α*-helices is an approximation and could lead to classification difficulties for the methodology. Furthermore, classification difficulties could arise from the arbitrary choice of window length made by the methodology as window length is known to have a profound effect on the relationship between <*μH*> and <*H*>[7]. It would seem that the optimisation of *θ *and window length are crucial to the classification of amphiphilic *α*-helices yet the values chosen for these parameters by hydrophobic moment plot analysis are not optimal for the classification of any single subclass.

A number of studies have considered the significance of <*μH*> in relation to structure / function relationships of the *α*-helical classes described by hydrophobic moment plot methodology with common examples including: surface active *α*-helices, transmembrane *α*-helices and oblique orientated *α*-helices [8-10]. However, if different *α*-helical classes have differing optima for *θ *and window length, not only does this question the validity of results obtained in these studies but also questions the validity of *α*-helix classification according to hydrophobic moment plot methodology. In this paper we examine the criteria upon which the methodology is based and, in view of their medical relevance, we use transmembrane *α*-helices as a test data set. These *α*-helices possess central regions, which are predominantly formed by hydrophobic residues and interact with the membrane lipid core, and end regions, which are primarily formed by hydrophilic residues and reside in the membrane surface regions [8]. For the *α*-helices of our data set, we analyse the relationships for the mean hydrophobic moment and window size, angular frequency and the robustness to varying angular frequency. Correlations between the mean hydrophobic moment and mean hydrophobicity of transmembrane *α*-helices are established, verified and analysed to appraise biological function using resampling Bootstrap and Monte Carlo techniques [11,12].

## Results

A data set of 84 transmembrane proteins were identified within Swiss-Prot and used to generate a set of 403 transmembrane sequences (see Additional file 1). All sequences within the data were of 21 residues in length and showed less than 5% homology (data not shown). For the sequences of this data set, the maximum mean hydrophobic moment, <*μH*>, and its corresponding mean hydrophobicity, <*H*>, were determined and used to generate the hydrophobic moment plot shown in figure 1, based on the generally used 11 residue window (*L *= 11) introduced by Eisenberg *et al*., [4]. It can be seen that data points representing the sequences of our data set cluster around the transmembrane region identified by Eisenberg *et al*., [5] but as previously noted [6] there are a significant number that fall outside the boundaries of this region. In particular, many of this number possess <*H*> values less than 0.5 and would not be classified as transmembrane *α*-helices according to the hydrophobic moment plot taxonomy of Eisenberg *et al*., [5]. Even allowing for the diffuse nature of these boundaries on the hydrophobic moment plot diagram [5], these results clearly question the efficacy of hydrophobic moment methodology for the prediction of transmembrane *α*-helices.

**Figure 2:**
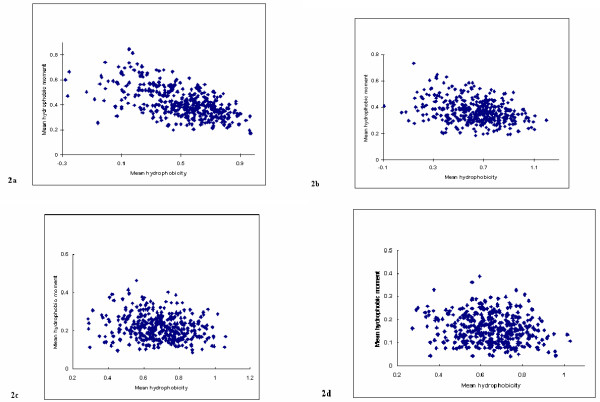
**Hydrophobic moment plot analysis of the transmembrane protein data set with varying window size. **Figure 2 shows the 403 transmembrane sequences of our data set, which were analysed according to hydrophobic moment plot methodology but with varying window size (L). In comparison to *L *= 1 (figure 1b), here in figure 2a, *L *= 7; in figure 2b, *L *= 9; in figure 2c, *L *= 16; and in figure 2d, *L *= 20. In each case, *θ *= 100°.

The above analysis was repeated except that window sizes varying in the range (7 ≤ *L *≤ 20) were employed. The values for <*μH*> and corresponding <*H*> were plotted as above and the results for window sizes of 7, 9, 16 and 20 are shown in figure 2. It can be seen that a weak negative correlation exists between <*μH*> and <*H*> for smaller window sizes but that the level of correlation appears to reduce as window size increases. The sample correlation coefficients for the various window sizes are given in table 1. To conduct standard statistical tests to determine whether the population correlation coefficients do differ from zero, it is necessary to establish if these data are bivariate Normal. The P-values obtained from Anderson-Darling and Kolmogorov-Smirnov tests for Normality for the various window sizes with *θ *= 100° are shown in table 2. These results present clear evidence that the populations for the variates for each window size are not bivariate Normal. These findings prompted the use of the bootstrap procedures to estimate the confidence intervals for the population correlation coefficient values for the window sizes in the range (7 ≤ *L *≤ 20).

**Table 1:** Sample correlation coefficients between <*μH*> and <*H*> for window sizes (7 ≤ *L *≤ 20).

**Window size (L)**	**Sample correlation coefficient (r)**	**Window size (L)**	**Sample correlation coefficient (r)**
**7**	-0.57648	**14**	-0.34654
**8**	-0.45020	**15**	-0.31280
**9**	-0.30316	**16**	-0.17998
**10**	-0.40110	**17**	-0.15074
**11**	-0.47663	**18**	-0.21843
**12**	-0.33693	**19**	-0.20038
**13**	-0.30354	**20**	-0.15653

**Table 2:** Confidence Intervals for regression coefficient from bivariate Normality goodness-of-fit for window size *L*. * 93% Confidence Interval

**Window size (L)**	**95% Confidence Interval**	**99% Confidence Interval**
**7**	(1.077, 1.112)	(1.072, 1.176)
**8**	(1.051, 1.084)	(1.046, 1.089)
**9**	(1.067, 1.091)	(1.061, 1.095)
**10**	(1.091, 1.171)	(1.078, 1.184)
**11**	(1.078, 1.134)	(1.068, 1.149)
**12**	(1.046, 1.075)	(1.042, 1.080)
**13**	(1.054, 1.110)	(1.047, 1.167)
**14**	(1.055, 1.124)	(1.044, 1.135)
**15**	(1.050, 1.087)	(1.044, 1.093)
**16**	(1.036, 1.045)	(1.030, 1.051)
**17**	(0.976, 1.001)	(0.977, 0.999)*
**18**	(0.959, 0.980)	(0.956, 0.983)
**19**	(0.957, 0.967)	(0.955, 0.968)
**20**	(0.950, 0.960)	(0.948, 0.962)

**Figure 3:**
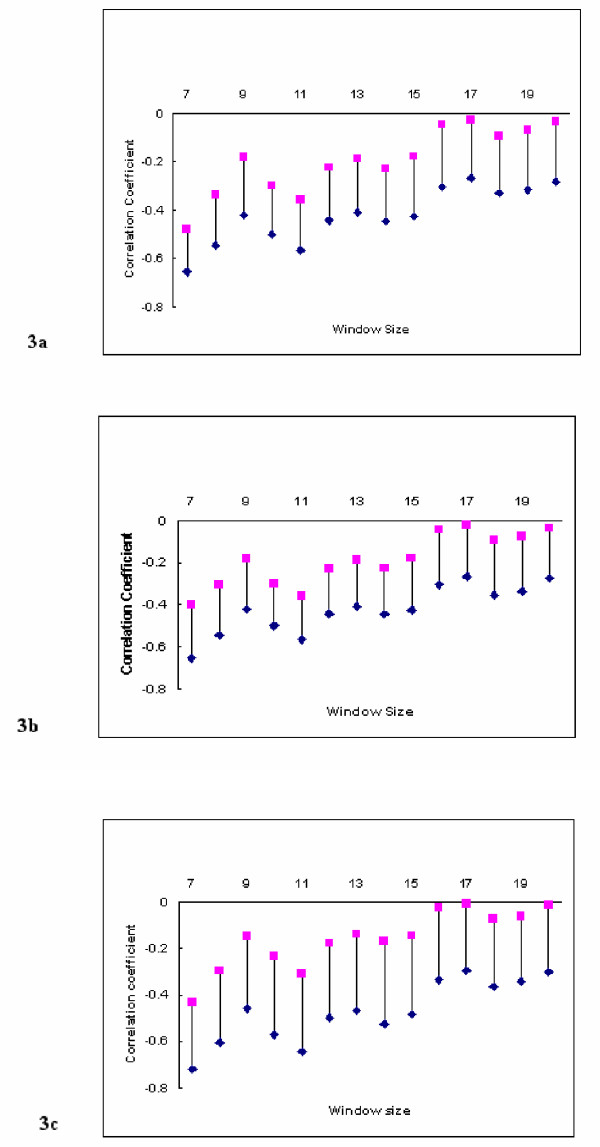
**Confidence intervals for the Correlation Coefficient. **Figure 3a shows the 99% BCa confidence intervals for the correlation coefficients estimated from 4000 bootstrap replicates. Figure 3b shows the 99% ABC confidence intervals for the correlation coefficients. Figure 3c shows the 99% Delta Method confidence intervals for the correlation coefficients.

**Figure 4:**
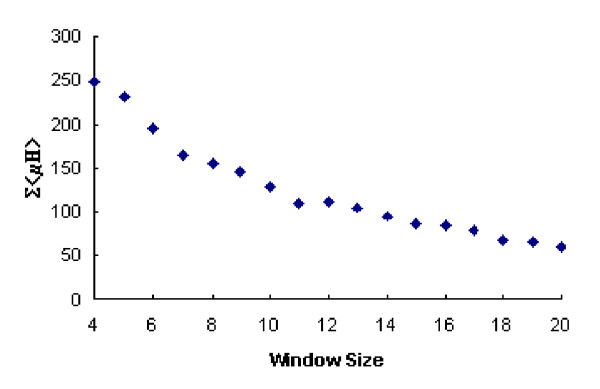
**Σ<*μH*> for the transmembrane protein data set for variable window sizes with optimised angular frequency. **Figure 4 shows the variation of Σ<*μH*> for the 403 transmembrane sequences of our data set with window size (7 ≤ *L *≤ 20) for optimised *θ *(95° ≤ *θ *≤ 108°).

**Figure 5:**
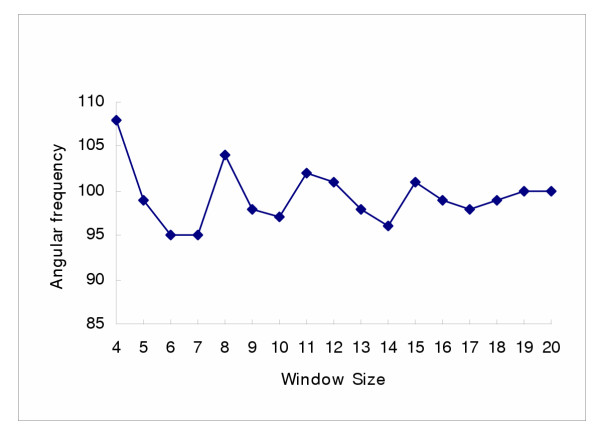
**The variation of optimal angular frequency with window size for the transmembrane protein data set. **Figure 5 shows the variation of optimal angular frequency, *θ*, (95° ≤ *θ *≤ 108°) with window size (7 ≤ *L *≤ 20) for the 403 transmembrane sequences ofour data set

**Figure 6:**
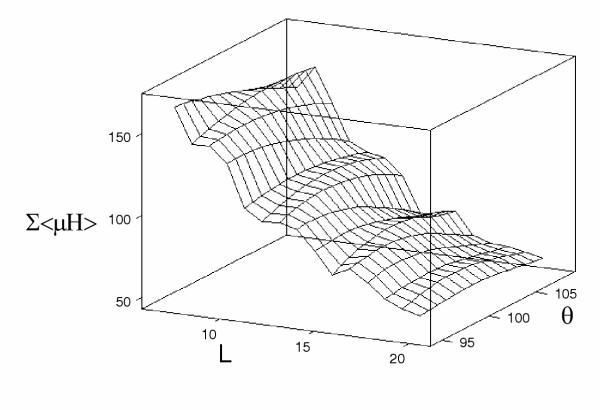
**Response surface diagram for the transmembrane protein data set. **Figure 6. Response surface diagram for the Σ<*μH*> for window sizes (7 ≤ *L *≤ 20) and angular frequency (95° ≤ *θ *≤ 108°).

The results of this investigation for *θ *= 100° are presented in figure 3. It would appear that the smaller window sizes do show correlations between <*μH*> and <*H*> and if this reflects a biological property of transmembrane sequences, it could be of use in the analysis and prediction of these motifs. It is known that angular frequency for a transmembrane *α*-helix varies between 95° and 107° [16], rather than being fixed at 100° as proposed by the methodology of Eisenberg *et al*., [4]. For each window size in the range (7 ≤ *L *≤ 21) residues, to accommodate the findings of Cornette *et al*., [16], the fixed value of *θ *was therefore varied from 95° to 108° in increments of 1°. Once the optimal window had been obtained, to observe the discriminating effect of *θ *on <*μH*>, the <*μH*> values, denoted by Σ<*μH*>, were summed for the 403 sequences for each *θ*. Figure 4 shows the optimal *θ*, based on the maximum values of Σ<*μH*> for each window length. It can be seen that as the window size increases the total <*μH*> reduces approximately linearly until the intermediate size of eleven residues in length. For subsequent larger window sizes, we observe a further near linear reduction trend but at a reduced rate. The optimal angular frequency corresponding to each window size (7 ≤ *L *≤ 21) is also given in figure 5. The overall relationship between Σ<*μH*>, the window size, *L*, and the angular frequency, *θ*, is finally depicted in figure 6 as a response surface diagram.

To assess the robustness of <*μH*> to this fixed angular frequency assumption, and thus, the accuracy of the hydrophobic moment plot analysis for candidate transmembrane sequences, Monte Carlo simulation studies were conducted. Initially, the angular frequency, *θ*, was assumed to have a mean value, E(*θ*), fixed at 100° and the angle for each successive residue varied about E(*θ*). The random variation, X, followed a Normal distribution and six separate simulations were undertaken with X~N(100, *σ*^2^), where the standard deviation, *σ*, was set at 0.1°, 0.3°, 0.5°, 0.7°, 0.9° and 1.1° respectively for each. The process was repeated with the mean value being set at the identified optimal angular frequency for the window size, again, for each of the window sizes in the range (7 ≤ *L *≤ 20).

**Figure 7:**
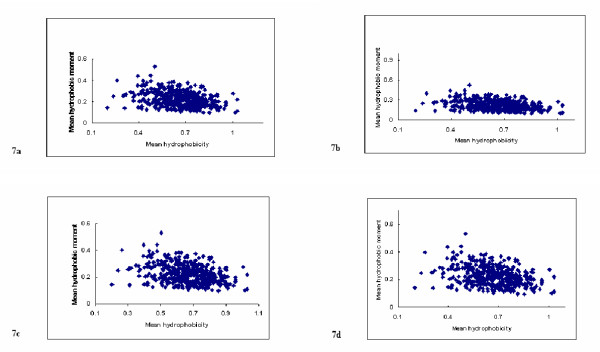
**Hydrophobic moment plot analysis of the transmembrane protein data set with varying standard deviation of *θ *about *θ *= 100°. **Figure 7 shows hydrophobic moment plot analysis of the 403 transmembrane sequences of our data set using *L *= 15 and: In figure 7a, *θ *= 100°; in figure 7b, *θ *is from a Normal Distribution with E(*θ*) = 100° and standard deviation of 0.1° ; In figure 7c, *θ *is from a Normal Distribution with E(*θ*) = 100° and standard deviation of 0.7° and in figure 7d, *θ *is from a Normal Distribution with E(*θ*) = 100° and standard deviation of 1.1°.

Hydrophobic moment plots for variable angular frequency were obtained for E(*θ*) = 100° for each window size in the range (7 ≤ *L *≤ 21) residues and for the separate standard deviation values, *σ *= 0.1°, 0.3°, 0.5°, 0.7°, 0.9°, 1.1°. These were compared visually with the original plots obtained under the fixed angular frequency assumption (*θ *= 100°). In all cases, the bulk properties of the plots were similar irrespective of the level of dispersion introduced by the different values of the standard deviation. The hydrophobic moment plot for *L *= 15; *θ *= 100° is provided in figure 7. This is to be contrasted with the plots for *L *= 15; E(*θ*) = 100°, *σ *= 0.1°, *σ *= 0.7° and *σ *= 1.1°, also present in figure 7. Similar results were obtained for all other values, confirming, at least visually, that <*μH*> is robust to slight random perturbations about a fixed value. These properties were also observed for the simulation study with the fixed angular frequency assumption being violated about the optimum frequency for each of the window sizes in the range (7 ≤ *L *≤ 20) and for each corresponding level of dispersion.

A more rigorous assessment of the variation was provided by analysis of the sample correlations. These were calculated in each case and compared to the empirically derived 99% confidence intervals established for window sizes in the range (7 ≤ *L *≤ 20) under the fixed angular frequency assumption of *θ *= 100°. The calculated sample correlation coefficients were also compared to the point estimates for the original data. In all cases, the values were within the appropriate confidence intervals and were always close to the original sample correlation coefficient values, again providing evidence that <*μH*> is robust to random variation in angular frequency. The results of this investigation are given in table 3.

**Table 3:** Sample correlation coefficients for optimum <*μH*> for *θ *= 100°, *θ*~N(100, *σ*^2^) and window sizes, *L *= 7, 11, 15, 16, 20.

**Window size (*L*)**	***θ *= 100; *σ *= 0**	***σ *= 0.1**	***σ *= 0.3**	***σ *= 0.5**	***σ *= 0.7**	***σ *= 0.9**	***σ *= 1.1**
**7**	-0.576465	-0.576557	-0.576118	-0.574907	-0.577803	-0.575951	-0.577435
**11**	-0.476666	-0.476109	-0.475923	-0.476820	-0.476131	-0.475736	-0.475371
**15**	-0.312882	-0.312924	-0.312973	-0.313221	-0.313488	-0.312796	-0.311120
**16**	-0.180014	-0.180160	-0.180679	-0.180656	-0.179292	-0.178218	-0.180065
**20**	-0.156516	-0.156837	-0.156606	-0.156546	-0.158868	-0.158272	-0.155921

**Figure 8:**
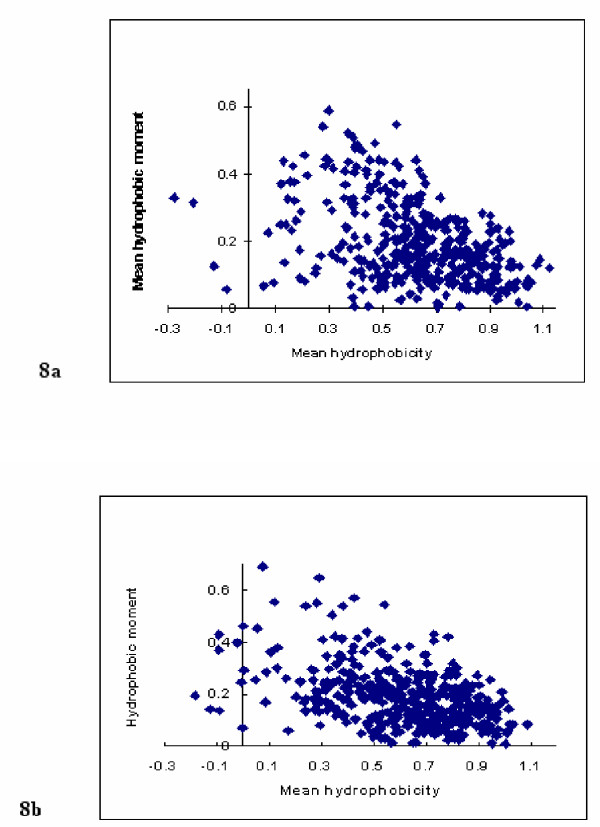
**Hydrophobic moment plot analysis of the transmembrane data set using randomised sequence arrangements. **Figure 8 Hydrophobic moment plot analysis of our data set was performed using sequences generated by a) random rearrangement of sequences for the optimal windows, b) random sequences formed with amino acid relative frequencies the same as those of the optimal windows. In all cases, *L *= 11 and *θ *= 100°.

To test whether these correlations are artefactual, hydrophobic moment plots were obtained for the <*μH*> and <*H*> derived from the 403 artificial randomisation sequences generated by random re-ordering or randomisation [20] of each of the original optimum window sequences. The plot for a window size of *L *= 11 is given in figure 8. These analyses were undertaken for all those window sizes with previously identified statistically significant correlation coefficients between <*μH*> and <*H*> and were designed to test the importance of the spatial arrangement of the amino acids within the optimum sequences.

Similar plots were obtained from Monte Carlo simulated data derived from the 403 sequences that had been generated by random sampling using the relative abundancies of residues found in the set of optimal windows. These analyses were therefore designed to look at the importance of relative amino acid composition for the correlations between <*μH*> and <*H*> and the results can be seen for a window size of *L *= 11 in figure 8. Again, analyses were performed for all window sizes with associated statistically significant correlations (data not shown). It is worth noting that since the effect of varying window size had a significant effect on the correlation between <*μH*> and <*H*>, varying *L *was observed to vary the optimal sequence identified within the transmembrane domain. Clearly this was not unexpected.

## Conclusions

It can be seen from figure 5 that the most discriminating angular frequency for a fixed window size varies within the range, (95° ≤ *θ *≤ 104°) for window sizes (7 ≤ *L *≤ 20). There is an obvious damped oscillation present, which can be seen to correspond to the assumed intrinsic periodicity of *α*-helical secondary structure i.e. 3.6 residues per turn. Figure 5 clearly demonstrates that the fixed 100° angular frequency, assumed when modelling *α*-helices in general, is no more than a representative average with a value nearer 102° providing a maximum for an optimum *L *= 11 residue window in a transmembrane *α*-helical sequence.

From figure 4, it is also evident that the degree of discrimination possible using <*μH*> declines in a near linear fashion with increasing window size with the optimum *L *= 11 residue window appearing to provide approximately average discrimination for transmembrane *α*-helices. The bootstrap derived 99% confidence intervals for the correlation coefficients between <*μH*> and <*H*> for window sizes in the range (7 ≤ *L *≤ 20) showed that there are highly significant linear associations for the smaller window sizes in the range (7 ≤ *L *≤ 16). As the magnitude of each of the corresponding sample coefficients is small (table 1), this should be interpreted as evidence of a strong (negative) association but with high variability being present. These correlations become weaker, on average, with increasing window size until they are not statistically significant at the 1% level and we have no compelling evidence that the variates are not independent. The choice of window size therefore, becomes paramount if <*H*> and <*μH*> are to be used to classify transmembrane *α*-helices. More importantly, the variation in correlation between these parameters and the effect of varying window size on the location of the sequence identified as optimal for *α*-helix classification brings into question the relevance of using the mean hydrophobic moment for comparison between varying window sizes. However, <*μH*> has been shown to be robust to departures from the fixed angular frequency assumption for a large range of window sizes appropriate for transmembrane proteins and for a range for levels of dispersion.

There were no substantial differences between the plots for relative abundance sample data and those for the randomisation sequences (figure 8) except for a few chance negative <*H*> observations from the former. This suggests that there are no serial correlations between residue types, where presence in the identified section of the penetrating transmembrane stretch is determined predominantly by relative abundance. This is to be contrasted with the distribution of observations for the original transmembrane sequences for a window size of 11 residues (figure 1). Most noticeable is the difference in <*μH*> over the range of <*H*> values. There appears to be a lower bound for <*μH*> for the original sequence, which is clearly not present for the randomisation data. Furthermore, whilst the negative correlation would appear to be an artefact, as it is exhibited in all cases, the dispersion around any optimal fitted line through the data such as a least squares fit also is clearly different. It appears similar and quite spread out for the two randomised sequence data but considerably less so for the transmembrane sequences. This provides evidence that within the optimum window, whilst residue composition is not influential, order is. It would appear that this ordering is leading to both organisation and biological function for at least segments of the interacting portions of transmembrane proteins. This is consistent with the belief that the hydrophobic moment is a good predictor of amphiphilicity [8] although it can be unduly influenced by relatively few amino acid residues within a sequence [21].

In summary, our analyses confirm previous studies, which have shown limitations to the ability of hydrophobic moment plot methodology to assign function to membrane interactive *α*-helices [6]. More importantly, our investigation leads to a questioning of the logic of comparing mean hydrophobic moments, in general, for transmembrane proteins. This is due to the effect of window size on both, the correlation of mean hydrophobic moment with mean hydrophobicity and the identified sensitivity of the optimum window. Comparisons of the hydrophobic moment are seemingly only meaningful for separate transmembrane proteins with identical window sizes.

Despite these limitations, <*μH*> has been shown to be robust to departures from the fixed angular frequency assumption for transmembrane proteins. Given the severe lack of structural information for transmembrane proteins, the identification of transmembrane *α*-helices using hydrophobic moment based analyses, and other bioinformatic approaches, seems likely to continue for the foreseeable future. Nonetheless, the results of such analyses should only be taken as a guide, and where possible, obtaining corroborative experimental data is essential. On the positive side, our results have demonstrated the importance of amino acid residue sequence order in establishing organisation and biological function for the transmembrane *α*-helices of proteins. With the ongoing development of predictive techniques, these results should be useful in furthering this development and helping to improve drug target identification.

## Methods

### The selection of transmembrane, *α*-helix forming segments

The primary structures of 96 transmembrane proteins were selected from the Swiss-Prot data bank (; accessed 25.05.04) and confirmed as transmembrane by extensive analysis of the literature. The sequences were analysed for homology using the sequence alignment program BLAST (Basic local alignment search tool) [13] and twelve homologous sequences were rejected. From the remaining 84 primary structures, a data set comprising 403 putative transmembrane *α*-helical sequences, each of 21 residues, was established using the algorithm, Top Pred2 ([14]; ; accessed 25.05.04).

### Hydrophobic moment plot analysis of transmembrane, *α*-helix forming segments

In the present study, all hydrophobic moment plot analyses were performed using the consensus hydrophobic scale of Eisenberg [4,5]. To identify putative transmembrane *α*-helix forming segments using hydrophobic moment plot methodology, hydropathy plot analysis [15] is initially undertaken to identify the primary amphiphilicity of candidate sequences. These sequences are selected using a 21 residue window as this is sufficiently long for an *α*-helix to span the bilayer.

Once a putative transmembrane domain has been identified, an eleven residue window is considered to progress along the amino acid sequence and for each window, the hydrophobic moment at 100° is calculated. Based on the assumption that a protein sequence will adopt its most amphiphilic arrangement, the window with the maximum mean hydrophobic moment, <*μH*>, is taken as the most likely to form an amphiphilic *α*-helix [5]. The location of the optimum window was observed accordingly for window sizes of seven through to twenty consecutive residues.

### Optimal angular frequency and window length for <*μH*>

For window sizes ranging from 7 to 20 amino acid residues <*μ*_*H*_> were computed for the range of angular frequency values (95° ≤ *θ *≤ 108°). In each case, the value of *θ*, which maximises <*μ*_*H*_>, i.e. the value of *θ *which produces <*μH*>, was determined and is referred to as the optimal angular frequency for that window size. These procedures were based on previously published work, which identified variations in *θ *for *α*-helices [16].

### Hydrophobic Correlation

For window sizes ranging from 7 to 20 amino acid residues, scatterplots of <*μH*> versus <*H*> (hydrophobic moment plots) with *θ *= 100° were obtained. The corresponding sample correlation coefficients were calculated to identify the effect of window size on the relationship between these variates and hence on their ability to act as discriminators in the prediction of transmembrane *α*-helices. In addition, for each window size in the range (7 ≤ *L *≤ 20) residues and for *θ *in the range (95° ≤ *θ *≤ 108°), the response surface diagram for <*μH*> was constructed.

### Confidence intervals for the Correlation Coefficient

Statistical confidence intervals were established for the Pearson (Product-Moment) Correlation Coefficient between <*μH*> and <*H*> for both cases where window size was varied for a fixed value of the angular frequency, and the angular frequency was varied for a fixed window size. The resulting mean hydrophobicity measures were checked for bivariate Normality and non-parametric bootstrap procedures [11] were used to estimate confidence intervals for the Correlation Coefficients [17].

To provide evidence of the statistical significance of any linear association, the bootstrap bias-corrected and accelerated technique (BCa) [18] and an analytical extension of this, the ABC [19]. In addition, the bootstrap Delta method was employed, which although another bootstrap based method, was developed specifically for estimating the variance of a function of sample means. As the sample Correlation Coefficient can be readily expressed as such a statistic, it is also well suited to the estimation of confidence intervals for these Correlation Coefficients [12]. As both main approaches differ substantially, a more informed assessment of statistical significance could therefore be made.

### Variable angular frequencies

To assess the robustness of <*μH*> to the fix angular frequency assumption, e.g., *θ *= 100°, *θ *was varied randomly about 100° and <*μH*> was calculated for each of the optimal windows for window sizes (7 ≤ *L *≤ 20) for the 403 transmembrane proteins. These calculations were also obtained for similar random variations about the observed optimum angular frequencies, again, for the various window sizes (7 ≤ *L *≤ 20). In all cases, it is assumed that the variation follows a Normal distribution with the mean value set at the desired value for *θ *and with the standard deviation, *σ*, set at: 0.1°, 0.3°, 0.5°, 0.7°, 0.9° and 1.1° respectively for six separate Monte Carlo simulation studies. The sample correlation coefficients for each simulation were calculated and compared to the empirically derived 99% confidence intervals for the corresponding population values and, in particular, with the point estimates for the original sequences.

### Causality and biological function

Given that these data are from an observational study, it is necessary to assess whether any linear associations between <*μH*> and <*H*> for the *α*-helix forming sequences of our data set are likely to be causal or merely an artefact of amino acid composition. To investigate these possibilities, two additional simulation studies were undertaken. The first looked at spatial arrangements of residues within the primary sequences and the second focused on the effect of amino acid composition on correlations between <*μH*> and <*H*>.

To assess if positional or sequential correlational properties existed for the amino acids within the sequences, the sequence of residues for each of the optimum windows was re-ordered randomly. Artificial sequences were thus generated by random rearrangement or randomisation [20] of the primary sequences within the 403 optimal windows. Hence, each window associated with <*μH*> was used to generate a random arrangement.

To further investigate whether correlations between <*μH*> and <*H*> were dependent on sequence composition and not on spatial or sequential correlation, an additional parametric bootstrap simulation study was conducted. Here 403 artificial sequences were created. Each was randomly generated where, for each position, selection was based on the relative abundance of all the residues for the complete 403 optimum windows.

In both cases the corresponding <*μH*> and <*H*> from these newly created sequences were calculated, the associated hydrophobic moment plots obtained and sample correlations calculated. These were inspected to assess whether any linear associations for the original transmembrane data were thus likely to be causal or merely artefactual and whether, from inspection of variation, there was evidence of increased organisation, which could be interpreted as an indication of biological function.

## Supplementary Material

Additional File 1 Transmembrane sequence data set Click here for file

## References

[B1] Brady AE, Limbird LE (2002). G protein-coupled receptor interacting proteins: Emerging roles in localization and signal transduction. Cellular Signalling.

[B2] Müller G (2000). Towards 3D structures of G protein-coupled receptors: a multidisciplinary approach. Curr Med Chem.

[B3] Phoenix DA, Harris F, Daman OA, Wallace J (2002). The prediction of amphiphilic *α*-helices. Curr Protein Pept Sci.

[B4] Eisenberg D, Weiss RM, Terwilliger TC (1982). The helical hydrophobic moment: a measure of the amphiphilicity of a helix. Nature.

[B5] Eisenberg D, Schwarz E, Komaromy M, Wall R (1984). Analysis of membrane and surface protein sequences with the hydrophobic moment plot. J Mol Biol.

[B6] Phoenix DA, Stanworth A, Harris F (1998). The hydrophobic moment plot and its efficacy in the prediction and classification of membrane interactive proteins and peptides. Membr Cell Biol.

[B7] Auger IE, Epand RM (1993). Computational techniques to predict amphipathic helical segments. The Amphipathic Helix.

[B8] Phoenix DA, Harris F (2002). The hydrophobic moment and its use in the classification of amphiphilic structures (Review). Mol Membr Biol.

[B9] Harris F, Wallace J, Phoenix DA (2000). Use of hydrophobic moment plot methodology to aid the identification of oblique orientated *α*-helices. Mol Membr Biol.

[B10] Phoenix DA, Harris F (2003). Is use of the hydrophobic moment a sound basis for predicting the structure-function relationships of membrane interactive *α*-helices?. Curr Protein Pept Sci.

[B11] Efron B, Tibshirani RJ (1993). An Introduction to the Bootstrap.

[B12] Efron B (1982). The Jackknife, the Bootstrap, and other Resampling Plans. Regional Conference Series in Applied Maths 38, SIAM, Philadelphia.

[B13] Altschul SF, Madden TL, Schaffer AA, Zhang J, Zhang Z, Miller W, Lipman DJ (1997). Gapped BLAST and PSI-BLAST: a new generation of protein database search programs. Nucleic Acids Res.

[B14] Von Heinje G (1992). Membrane-protein structure prediction-hydrophobicity analysis and the positive-inside rule. J Mol Biol.

[B15] Kyte J, Dolittle RF (1982). A simple method for displaying the hydropathic character of a protein. J Mol Biol.

[B16] Cornette JL, Cease KB, Margelit H, Spouge JL, Berzofsky JA, De Lisi CD (1987). Hydrophobicity scales and computational techniques for detecting amphipathic structures in proteins. J Mol Biol.

[B17] Sprent P (1998). Data driven Statistical Methods.

[B18] Buckland ST (1983). Monte Carlo methods for confidence interval estimation using the bootstrap technique. Bull Appl Statist.

[B19] Efron B, DiCiccio T (1992). More accurate confidence intervals in exponential families. Biometrika.

[B20] Manly BFJ (1991). Randomization and Monte Carlo Methods in Biology.

[B21] Pewsey AR, Phoenix DA, Roberts MG (1996). Monte Carlo analysis of potential C-terminal membrane interactive *α*-helices. Prot Pep Lett.

