# Supplementary material for: Investigation of hydrophobic moment and hydrophobicity properties for transmembrane α-helices
Source: Theor Biol Med Model. 2004 Aug 16;1:5. doi: 10.1186/1742-4682-1-5 (PMC516255; doi:10.1186/1742-4682-1-5)
Supplement: Additional File 1 — Transmembrane sequence data set [file 1742-4682-1-5-S1.doc]

**Protein Sequence Start End**

| ALLSD | Gluco | QIRLLVATTAVTALLVAAGYR* | 5 | 25 |
| --- | --- | --- | --- | --- |
| APALDH | AvaI | RGFLVTSLGAGVMFGFARPSS* | 23 | 43 |
| APALDH | AvaI | PKWGLMVTGGSWSVWMTWDVF* | 115 | 135 |
| AVAJ3195 | devB | IWWGLAVALPVAVAAGLLATA* | 22 | 42 |
| AVAJ3195 | devC | LAVALAGIAFADILMFMQIGF* | 20 | 40 |
| AVAJ3195 | devC | AIGFIFTLGTIMGFIVGTVIV* | 260 | 280 |
| AVAJ3195 | devC | LLLACLGYLPGWVFAMVLYQT* | 317 | 337 |
| AVAJ3195 | devC | AITVLILTMIMCFVSGSIAVR* | 353 | 373 |
| BACAPABC | capB | MIFIIGICTVFLIIYGIWEQR* | 1 | 21 |
| BACAPABC | capC | FGSDLYIALVLGVTLSLIFTE* | 2 | 22 |
| BACAPABC | capC | PVFMLVVLFISILTYVIVTYG* | 44 | 64 |
| BACAPABC | capC | PVFMLVVLFISILTYVIVTYG* | 44 | 64 |
| BACAPABC | capC | GLPLTIGTTILLSGATFAIMN* | 124 | 144 |
| BACAPABC | capA | RYVAIVLPLIAVILIAATWVQ* | 24 | 44 |
| BFJCG | pf1geneIII | VFAFLLYVATFMYVFSTFANI* | 397 | 417 |
| BJCYTC | cycM | ILGAVLGTCLILLVTSFTANA* | 9 | 29 |
| BJTLPAGN | tlpA | IPLVIATVAVGGLAGFAALYG* | 12 | 32 |
| BSCSBA | csbA | MITKAVFALFFPFMLVVLFTR* | 1 | 21 |
| BSCSBA | csbA | FNHYVAIALTAALLFASYLKG* | 24 | 44 |
| BSCSBA | csbA | ETYFIVGLDVVSLVAGGLYMA* | 47 | 67 |
| BSNRGABO | nrgA | TVFMFFCALLVWLMTPGLALF* | 6 | 26 |
| BSNRGABO | nrgA | SFSSIAIVSIVWVLFGYTLAF* | 43 | 63 |
| BSNRGABO | nrgA | SLFMMFQMTFAVLTTAIISGA* | 95 | 115 |
| BSNRGABO | nrgA | FGAFLLFSVLWASLVYTPVAH* | 122 | 142 |
| BSNRGABO | nrgA | GNVVHISSGVAGLVLAIVLGK* | 161 | 181 |
| BSNRGABO | nrgA | LIYTFLGGALIWFGWFGFNVG* | 193 | 213 |
| BSNRGABO | nrgA | NTNTAAAAGIAGWILVEWIIN* | 228 | 248 |
| BSNRGABO | nrgA | PTMGAVSGAIAGLVAITPAAG* | 251 | 271 |
| BSNRGABO | nrgA | ASIIIGIIGGAVCFWGVFSLK* | 277 | 297 |
| BSNRGABO | nrgA | HGIGGTWGGIATGLFATTSVN* | 312 | 332 |
| BSNRGABO | nrgA | IVAIAATYVFVFIVTFVIIKI* | 351 | 371 |
| BSSPOII | spoIIGA | VIWLLNFCFDALLLLLTAFIL* | 7 | 27 |
| BSSPOII | spoIIGA | LVGGAFIGSSIVLLMFTPFSP* | 36 | 56 |
| BSSPOII | spoIIGA | HPAGKLAFSVVIVVVTFGFKR* | 60 | 80 |
| BSSPOII | spoIIGA | LFSFYFATFLMGGGIIGAHSL* | 88 | 108 |
| BSSPOII | spoIIGA | GDPISWLFIVGGFPALWFFSK* | 128 | 148 |
| CCMCPA | mcpA | GFAPAFALLMLAVMAGGAILV* | 16 | 36 |
| CCMCPA | mcpA | TVSGMISVDFNMAAGFIAPFE* | 137 | 157 |
| CCMCPA | mcpA | IIMSLLTLGAVGALAFLTVMT* | 197 | 217 |
| CFCAI01 | colA | ALYLFIIIGLIPLLCIFVVYY* | 17 | 37 |
| CFCAI01 | colA | IYCKTAPFLALILYILTFKIR* | 72 | 92 |
| CFCAI01 | colA | LRSCLLSPLVYAAIVYLFCFR* | 104 | 124 |
| CFCAI01 | colA | ATLLLFYIGLYSIIFFTTYIT* | 143 | 163 |
| CGPUTP | putP | TWFIIAIVIYMLVMVLIGYWS* | 5 | 25 |
| CGPUTP | putP | MSGWLLMGLPGALFVTGMSEL* | 56 | 76 |
| CGPUTP | putP | IIAALIIIVFFTFYISSGMVA* | 127 | 147 |
| CGPUTP | putP | LLGMAIVAGVTVLYTFIGGFL* | 161 | 181 |
| CGPUTP | putP | QGTIMFFSLIIVPVMAYFALA* | 190 | 210 |
| CGPUTP | putP | PTYFSMISGISAAAIIGNLGG* | 234 | 254 |
| CGPUTP | putP | SWMIICLIGATFTAIISTVFF* | 285 | 305 |
| CGPUTP | putP | LIAGLILTAVLAAIMSTMSSQ* | 334 | 354 |
| CGPUTP | putP | SRATVIILAIIAAAMAINPSD* | 384 | 404 |
| CGPUTP | putP | LGLVGFAWAGFGSAFGPIILA* | 407 | 427 |
| CGPUTP | putP | AGAISGMITGAIVSIAWGMSP* | 437 | 457 |
| CGPUTP | putP | IIPGFALATIVMVVVSLLTKE* | 465 | 485 |
| CPPBG | ORF54 | SLSSGALMLMTFTAVFSFGNI* | 7 | 27 |
| CPPBG | ORF54 | TIPSYIFGTVFYFLPFALMIG* | 38 | 58 |
| CPPBG | ORF54 | WAFLGSWSYFFVNLFFFTSLL* | 83 | 103 |
| CPPBG | ORF54 | KTVLISVISIVLFWAVTIIST* | 123 | 143 |
| CPPBG | ORF54 | IILGLGFIVLSFGVILFLGKA* | 161 | 181 |
| CPPBG | ORF54 | FNWSYFMVLAWILQAVGGAES* | 195 | 215 |
| CPPBG | ORF54 | TMVISTAIVGGLYALGAVSVG* | 233 | 253 |
| CPPBG | ORF54 | VGFIMMLASLGSLVLWTAAPV* | 289 | 309 |
| CPPBG | ORF54 | VQAVIVTVLLLVPALGIGSVD* | 340 | 360 |
| CPPBG | ORF54 | MTASTSLIPVLFFLVGYIVLR* | 369 | 389 |
| CPPBG | ORF54 | IAIGVLLLALFVFVFVISSIP* | 408 | 428 |
| CPPBG | ORF54 | NPVFILLYNVLGLVFFLGFAE* | 447 | 467 |
| CTOMP3D | chltr | DVIPLSLGIETLGGVMTPLVE* | 382 | 402 |
| CVCYCA | cycA | LAIGGAVLMGTLFFLVSFLTG* | 12 | 32 |
| CVCYCA | cycA | SFMGWFLLIFCASLIIMGLGK* | 48 | 68 |
| CVCYCA | cycA | KWFLSFPLSIFVIVMVMFFSL* | 76 | 96 |
| CVPBR322 | tecr2Ec | ALIVILGTVTLDAVGIGLVMP* | 6 | 26 |
| CVPBR322 | tecr2Ec | GVLLALYALMQFLCAPVLGAL* | 46 | 66 |
| CVPBR322 | tecr2Ec | PVLLASLLGATIDYAIMATTP* | 74 | 94 |
| CVPBR322 | tecr2Ec | IVAGITGATGAVAGAYIADIT* | 104 | 124 |
| CVPBR322 | tecr2Ec | FGLMSACFGVGMVAGPVAGGL* | 133 | 153 |
| CVPBR322 | tecr2Ec | APFLAAAVLNGLNLLLGCFLM* | 161 | 181 |
| CVPBR322 | tecr2Ec | GMTIVAALMTVFFIMQLVGQV* | 208 | 228 |
| CVPBR322 | tecr2Ec | WSATMIGLSLAFGILHALAQA* | 243 | 263 |
| CVPBR322 | tecr2Ec | QAIIAGMAADALGYVLLAFAT* | 277 | 297 |
| CVPBR322 | tecr2Ec | WMAFPIMILLASGGIGMPALQ* | 300 | 320 |
| CVPBR322 | tecr2Ec | LAALTSLTSITGPLIVTAIYA* | 339 | 359 |
| CVPBR322 | tecr2Ec | WNGLAWIVGAALYLVCLPALR* | 365 | 385 |
| ECACRAB | acrB | PIFAWVIAIIIMLAGGLAILK* | 9 | 29 |
| ECACRAB | acrB | TLVEAIILVFLVMYLFLQNFR* | 342 | 362 |
| ECACRAB | acrB | AVPVVLLGTFAVLAAFGFSIN* | 370 | 390 |
| ECACRAB | acrB | TMFGMVLAIGLLVDDAIVVVE* | 393 | 413 |
| ECACRAB | acrB | LVGIAMVLSAVFVPMAFFGGS* | 441 | 461 |
| ECACRAB | acrB | SITIVSAMALSVLVALILTPA* | 470 | 490 |
| ECACRAB | acrB | VLYLIIVVGMAYLFVRLPSSF* | 541 | 561 |
| ECACRAB | acrB | SLYAISLIVVFLCLAALYESW* | 872 | 892 |
| ECACRAB | acrB | VMLVVPLGVIGALLAATFRGL* | 898 | 918 |
| ECACRAB | acrB | MTSLAFILGVMPLVISTGAGS* | 974 | 994 |
| ECACRAB | acrB | VTATLAIFFVPVFFVVVRRRF* | 1009 | 1029 |
| ECARAEA | araEEc | MNMFVSVAAAVAGLLFGLDIG* | 21 | 41 |
| ECARAEA | araEEc | WVVSSMMLGAAIGALFNGWLS* | 63 | 83 |
| ECARAEA | araEEc | LMAGAILFVLGSIGSAFATSV* | 92 | 112 |
| ECARAEA | araEEc | ARVVLGIAVGIASYTAPLYLS* | 118 | 138 |
| ECARAEA | araEEc | GKMISMYQLMVTLGIVLAFLS* | 147 | 167 |
| ECARAEA | araEEc | AMLGVLALPAVLLIILVVFLP* | 179 | 199 |
| ECARAEA | araEEc | VFLGMLLQAMQQFTGMNIIMY* | 258 | 278 |
| ECARAEA | araEEc | ATLVVGLTFMFATFIAVFTVD* | 298 | 318 |
| ECARAEA | araEEc | IGFSVMALGTLVLGYCLMQFD* | 328 | 348 |
| ECARAEA | araEEc | GLSWLSVGMTMMCIAGYAMSA* | 355 | 375 |
| ECARAEA | araEEc | TTTNWVSNMIIGATFLTLLDS* | 400 | 420 |
| ECARAEA | araEEc | FWLYTALNIAFVGITFWLIPE* | 427 | 447 |
| ECCEA | imm1Ec | YIKNILFGLYCTLIYIYLITK* | 6 | 26 |
| ECCEA | imm1Ec | LNLFMLYNLLCLVLAIPFGLL* | 84 | 104 |
| ECCET | Cet | FWKMTSLFGAVLLLLIPIMLI* | 7 | 27 |
| ECCET | Cet | AILLIALTFMAFFVFETLTAQ* | 298 | 318 |
| ECCET | Cet | QYLLVGLSLVMFYLLLLALSE* | 324 | 344 |
| ECCET | Cet | GFTVAWIIASLIGAIMNGIYL* | 347 | 367 |
| ECCET | Cet | NSMLFTLALLLLDGVMWGLLN* | 376 | 396 |
| ECCET | Cet | ALLLGTSVLVVALAGMMFVTR* | 401 | 421 |
| ECCYD | cyd | AMYHFLFVPLTLGMAFLLAIM* | 16 | 36 |
| ECCYD | cyd | FWGKLFGINFALGVATGLTME* | 54 | 74 |
| ECCYD | cyd | EGLMAFFLESTFVGLFFFGWD* | 99 | 119 |
| ECCYD | cyd | MCVTWLVALGSNLSALWILVA* | 127 | 147 |
| ECCYD | cyd | VASGYVTGAMFILGISAWYML* | 188 | 208 |
| ECCYD | cyd | SFAIAASFGMAAVLSVIVLGD* | 219 | 239 |
| ECCYD | cyd | MVACGFLLLAIIALSFWSVIR* | 392 | 412 |
| ECCYD | cyd | ALYGIPLPWIAVEAGWFVAEY* | 425 | 445 |
| ECCYD | cyd | IFSMVLICGLYTLFLVAELFL* | 471 | 491 |
| ECDMS | dmsC | LMIFTVFGQCVAGGFIVLALA* | 10 | 30 |
| ECDMS | dmsC | VIACMFGLWVLMGIGFIASML* | 44 | 64 |
| ECDMS | dmsC | IASGSIFFAVGGIGWLLAMLK* | 88 | 108 |
| ECDMS | dmsC | TLWLIVTMVLGVIFVWMMVRV* | 116 | 136 |
| ECDMS | dmsC | TPMGFFLTMFMGGPLLGYLLL* | 151 | 171 |
| ECDMS | dmsC | LLPAISVLALVVSGVVSVMQG* | 183 | 203 |
| ECDMS | dmsC | LMSWRIVLLAVALCLWIAPQL* | 226 | 246 |
| ECDMS | dmsC | QPAVLLSVSFILLLAGELIGR* | 250 | 270 |
| ECEXBBD | exbbEc | VKCVMIGLILASVVTWAIFFS* | 23 | 43 |
| ECEXBBD | exbbEc | LATIGAISPFVGLFGTVWGIM* | 133 | 153 |
| ECEXBBD | exbbEc | ALLATAIGLVAAIPAVVIYNV* | 177 | 197 |
| ECFTSQA | ftsA | LAGILFLLTVLTTVLVSGWVV* | 25 | 45 |
| ECFUCOSE | fucpEc | SYIIPFALLCSLFFLWAVANN* | 23 | 43 |
| ECFUCOSE | fucpEc | LIQSAFYFGYFIIPIPAGILM* | 65 | 85 |
| ECFUCOSE | fucpEc | KAGIITGLFLYALGAALFWPA* | 91 | 111 |
| ECFUCOSE | fucpEc | IMNYTLFLVGLFIIAAGLGCL* | 114 | 134 |
| ECFUCOSE | fucpEc | FNSFGAIIAVVFGQSLILSNV* | 161 | 181 |
| ECFUCOSE | fucpEc | TPYMIIVAIVLLVALLIMLTK* | 210 | 230 |
| ECFUCOSE | fucpEc | MTAGFAANYLTGTMVCFFIGR* | 291 | 311 |
| ECFUCOSE | fucpEc | LAAYALIAMALCLISAFAGGH* | 327 | 347 |
| ECFUCOSE | fucpEc | LIALTLCSAFMSIQYPTIFSL* | 350 | 370 |
| ECFUCOSE | fucpEc | SFIVMTIIGGGIVTPVMGFVS* | 384 | 404 |
| ECFUCOSE | fucpEc | PTAELIPALCFAVIFIFARFR* | 411 | 431 |
| ECGLPRZ | glpG | GPVTWVMMIACVVVFIAMQIL* | 94 | 114 |
| ECGLPRZ | glpG | ALMHFSLMHILFNLLWWWYLG* | 142 | 162 |
| ECGLPRZ | glpG | FTGPWFGGLSGVVYALMGYVW* | 192 | 212 |
| ECGLPRZ | glpG | GLIIFALIWIVAGWFDLFGMS* | 228 | 248 |
| ECGLPRZ | glpG | NGAIAGLAVGLAMAFVDSLNA* | 251 | 271 |
| ECGLYWA | pgsA | VILIPFFVLVFYLPVTWSPFA* | 14 | 34 |
| ECGLYWA | pgsA | YAGIALFFVAAVLTLWSMLQY* | 151 | 171 |
| ECLAC | lacYEc | TNFWMFGLFFFFYFFIMGAYF* | 7 | 27 |
| ECLAC | lacYEc | TGIIFAAISLFSLLFQPLFGL* | 45 | 65 |
| ECLAC | lacYEc | WIITGMLVMFAPFFIFIFGPL* | 78 | 98 |
| ECLAC | lacYEc | NILVGSIVGGIYLGFCFNAGA* | 102 | 122 |
| ECLAC | lacYEc | RMFGCVGWALCASIVGIMFTI* | 144 | 164 |
| ECLAC | lacYEc | QFVFWLGSGCALILAVLLFFA* | 167 | 187 |
| ECLAC | lacYEc | QPKLWFLSLYVIGVSCTYDVF* | 219 | 239 |
| ECLAC | lacYEc | VTTMGELLNASIMFFAPLIIN* | 263 | 283 |
| ECLAC | lacYEc | LLLAGTIMSVRIIGSSFATSA* | 291 | 311 |
| ECLAC | lacYEc | YLVCFCFFKQLAMIFMSVLAG* | 349 | 369 |
| ECLAC | lacYEc | LVLGLVALGFTLISVFTLSGP* | 382 | 402 |
| ECLCTPRD | lctP | IWLSSLIASLPILFFFFALIK* | 14 | 34 |
| ECLCTPRD | lctP | GYVAASWTVAIALAVALLFYK* | 39 | 59 |
| ECLCTPRD | lctP | YGFFYGLWPIAWIIIAAVFVY* | 71 | 91 |
| ECLCTPRD | lctP | GAAGFGAPVAITAALLVGLGF* | 131 | 151 |
| ECLCTPRD | lctP | TAPVAFGAMGIPILVAGQVTG* | 165 | 185 |
| ECLCTPRD | lctP | RQLPFMTIIVLFWIMAIMDGW* | 197 | 217 |
| ECLCTPRD | lctP | ETWPAVVVAGGSFAIAQYLSS* | 222 | 242 |
| ECLCTPRD | lctP | ELPDIISLVSLLCLTLFLKRW* | 248 | 268 |
| ECLCTPRD | lctP | WTPFLFLTATVTLWSIPPFKA* | 302 | 322 |
| ECLCTPRD | lctP | FSATGTAILFAALLSIVWLKM* | 368 | 388 |
| ECLCTPRD | lctP | ALPIYSIGMVLAFAFISNYSG* | 405 | 425 |
| ECLCTPRD | lctP | HAFTFFSPFLGWLGVFLTGSD* | 439 | 459 |
| ECLCTPRD | lctP | KMISPQSIAIAAAVGLVGKES* | 496 | 516 |
| ECLCTPRD | lctP | IFTCIVGVITTLQAYVLTWMI* | 528 | 548 |
| ECLEP | lep | ANMFALILVIATLVTGILWCV* | 2 | 22 |
| ECLEP | lep | LETGASVFPVLAIVLIVRSFI* | 60 | 80 |
| ECLEP | lep | PFQIPSGSMMPTLLIGDFILV* | 83 | 103 |
| ECLIPEP | lspAEc | SICSTGLRWLWLVVVVLIIDL* | 4 | 24 |
| ECLIPEP | lspAEc | RWFFAGIAIGISVILAVMMYR* | 69 | 89 |
| ECLIPEP | lspAEc | LADTAICVGAALIVLEGFLPS* | 139 | 159 |
| ECLIT | gplit protein | VLTVASLEYIWAFSNFFWVFT* | 62 | 82 |
| ECMALB | malF | WSVLGLLGLLVGYLVVLMYAQ* | 17 | 37 |
| ECMALB | malF | YLFAITTLILSSAGLYIFANR* | 40 | 60 |
| ECMALB | malF | MAGMGLFVLFPLVCTIAIAFT* | 72 | 92 |
| ECMALB | malF | FLAIFVWTVVFSLITVFLTVA* | 276 | 296 |
| ECMALB | malF | LLILPYAVPSFISILIFKGLF* | 319 | 339 |
| ECMALB | malF | IIVNTWLGYPYMMILCMGLLK* | 372 | 392 |
| ECMALB | malF | TLPLLIKPLTPLMIASFAFNF* | 417 | 437 |
| ECMALB | malF | GLAAAIATLIFLLVGALAIVN* | 484 | 504 |
| ECMDOGH | MdoH | YILLILTLAQTVVATWYMKTI* | 139 | 159 |
| ECMDOGH | MdoH | TGILILFAVLFCWVSAGFWTA* | 192 | 212 |
| ECMDOGH | MdoH | TGVMSYLSAPLWFMFLALSTA* | 514 | 534 |
| ECMDOGH | MdoH | LAIALFASTMVLLFLPKLLSI* | 564 | 584 |
| ECMDOGH | MdoH | MLFHTVFVVSAFLGWEVVWNS* | 620 | 640 |
| ECMDOGH | MdoH | HGSQLLLGLVWAVGMAWLDLR* | 657 | 677 |
| ECMDOGH | MdoH | FWLAPIVFSLILSPFVSVISS* | 680 | 700 |
| ECMOTAB | motA | MLILLGYLVVLGTVFGGYLMT* | 1 | 21 |
| ECMOTAB | motA | PAELVIIAGAGIGSFIVGNNG* | 31 | 51 |
| ECMOTAB | motA | SLPAFGIVAAVMGVVHALGSA* | 171 | 191 |
| ECMOTAB | motA | AMVGTFLGILLAYGFISPLAT* | 205 | 225 |
| ECMOTAB | motB | DFMTAMMAFFLVMWLISISSP* | 32 | 52 |
| ECOMPB | envZEc | TLLLIVTLLFASLVTTYLVVL* | 15 | 35 |
| ECOMPB | envZEc | RYTLAIMLLAIGGAWLFIRIQ* | 162 | 182 |
| ECPBPA | pbpA | RALVAFLGILLLTGVLIANLY* | 20 | 40 |
| ECPHOM | 17 kd protein | YKHLILSLSLIMLGPLAHAEE* | 3 | 23 |
| ECPHOM | ORF4 | FWKMTSLFGAVLLLLIPIMLI* | 7 | 27 |
| ECPHOM | ORF4 | AILLIALTFMAFFVFETLTAQ* | 298 | 318 |
| ECPHOM | ORF4 | QYLLVGLSLVMFYLLLLALSE* | 324 | 344 |
| ECPHOM | ORF4 | GFTVAWIIASLIGAIMNGIYL* | 347 | 367 |
| ECPHOM | ORF4 | NSMLFTLALLLLDGVMWGLLN* | 376 | 396 |
| ECPHOM | ORF4 | ALLLGTSVLVVALAGMMFVTR* | 401 | 421 |
| ECPONB | pbpBCc | GWLWLLLKLAIVFAVLIAIYG* | 64 | 84 |
| ECPTSG | ptsG | SLMLPVSVLPIAGILLGVGSA* | 15 | 35 |
| ECPTSG | ptsG | GGSVFANMPLIFAIGVALGFT* | 52 | 72 |
| ECPTSG | ptsG | ALAAVVAYGIMVKTMAVVAPL* | 79 | 99 |
| ECPTSG | ptsG | LADTGVLGGIISGAIAAYMFN* | 113 | 133 |
| ECPTSG | ptsG | FVPIISGLAAIFTGVVLSFIW* | 152 | 172 |
| ECPTSG | ptsG | LSGGLFKMYGLPAAAIAIWHS* | 250 | 270 |
| ECPTSG | ptsG | KVGGIMISAALTSFLTGITEP* | 278 | 298 |
| ECPTSG | ptsG | YIIHAILAGLAFPICILLGMR* | 311 | 331 |
| ECPTSG | ptsG | KLWLFPIVGIGYAIVYYTIFR* | 352 | 372 |
| ECPTSG | ptsG | GAAGVVVAGSGVQAIFGTKSD* | 443 | 463 |
| ECRHAT1 | rhaTEc | AITMGIFWHLIGAASAACFYA* | 4 | 24 |
| ECRHAT1 | rhaTEc | SVGGIVSWIILPWAISALLLP* | 39 | 59 |
| ECRHAT1 | rhaTEc | LPVFLFGAMWGIGNINYGLTM* | 74 | 94 |
| ECRHAT1 | rhaTEc | LGMSMGIGIAIGITLIVGTLM* | 97 | 117 |
| ECRHAT1 | rhaTEc | GRMTLLGVLVALIGVGIVTRA* | 134 | 154 |
| ECRHAT1 | rhaTEc | GLVLAVMCGIFSAGMSFAMNA* | 174 | 194 |
| ECRHAT1 | rhaTEc | LPSYVVIMGGGAIINLGFCFI* | 214 | 234 |
| ECRHAT1 | rhaTEc | LLSTLGGLMWYLQFFFYAWGH* | 259 | 279 |
| ECRHAT1 | rhaTEc | SWMLHMSFYVLCGGIVGLVLK* | 290 | 310 |
| ECRHAT1 | rhaTEc | VTVLSLGCVVIIVAANIVGIG* | 320 | 340 |
| ECSECDF | secD | YVMLIVVIVIGLLYALPNLFG* | 10 | 30 |
| ECSECDF | secD | LEACLAGLLVSILFMIIFYKK* | 453 | 473 |
| ECSECDF | secD | ILIVGIMSLLPGATLSMPGIG* | 487 | 507 |
| ECSECDF | secD | AITTGIGVATSMFTAIVGTRA* | 577 | 597 |
| ECSECDF | secF | YWAFGISGLLLIAAIVIMGVR* | 24 | 44 |
| ECSECDF | secF | AQTGAMALMAALLSILVYVGF* | 143 | 163 |
| ECSECDF | secF | AGVVIALAHDVIITLGILSLF* | 171 | 191 |
| ECSECDF | secF | ITSGTLMVILMLYLFGGPVLE* | 250 | 270 |
| ECSECDF | secF | SLTMLIGVSIGTASSIYVASA* | 273 | 293 |
| ECSECE | secEEc | AMKWVVVVALLLVAIVGNYLY* | 16 | 36 |
| ECSECE | secEEc | ALAVVILIAAAGGVALLTTKG* | 45 | 65 |
| ECSECE | secEEc | LHTTLIVAAVTAVMSLILWGL* | 91 | 111 |
| ECSECGLE | secG | EALLVVFLIVAIGLVGLIMLQ* | 3 | 23 |
| ECSECGLE | secG | DMGASFGAGASATLFGSSGSG* | 29 | 49 |
| ECSECGLE | secG | RMTALLATLFFIISLVLGNIN* | 54 | 74 |
| ECSPC | secYEc | LLFVIGALIVFRIGSFIPIPG* | 23 | 43 |
| ECSPC | secYEc | ASIFALGIMPYISASIIIQLL* | 75 | 95 |
| ECSPC | secYEc | YGTLVLAIFQSIGIATGLPNM* | 122 | 142 |
| ECSPC | secYEc | AFYFTAVVSLVTGTMFLMWLG* | 155 | 175 |
| ECSPC | secYEc | GNGISIIIFAGIVAGLPPAIA* | 184 | 204 |
| ECSPC | secYEc | FLVLLLVAVLVFAVTFFVVFV* | 217 | 237 |
| ECSPC | secYEc | AGVIPAIFASSIILFPATIAS* | 271 | 291 |
| ECSPC | secYEc | VLLYASAIIFFCFFYTALVFN* | 317 | 337 |
| ECSPC | secYEc | RLTLVGALYITFICLIPEFMR* | 371 | 391 |
| ECSPC | secYEc | PFYFGGTSLLIVVVVIMDFMA* | 397 | 417 |
| ECTOLQRA | tolREc | DVLLVLLLIFMATAPIITQSV* | 23 | 43 |
| ECTONB | tonBEc | FPWPTLLSVCIHGAVVAGLLY* | 14 | 34 |
| ECTRASTD | TraSp | PGLIIMGWMIFCPLLMSFLIT* | 34 | 54 |
| ECTRASTD | TraSp | TLTAVLAGGWLGLIILFIVAR* | 60 | 80 |
| ECTRASTD | TraSp | VYFIVYMGVIFLWSFLGGGII* | 108 | 128 |
| ECUGP | ugpA | PYLLVAPQLIITVIFFIWPAG* | 14 | 34 |
| ECUGP | ugpA | FSTFVTVSGLLVSLFFAALVE* | 78 | 98 |
| ECUGP | ugpA | LMLLPYAVAPAVAAVLWIFLF* | 110 | 130 |
| ECUGP | ugpA | IALPLIAPVSFFLLVVNLVYA* | 206 | 226 |
| ECUGP | ugpA | SAAQSVVLMFLVIVLTVVQFR* | 265 | 285 |
| ECUGP | ugpE | TMLILGIAVILFPLYVAFVAA* | 14 | 34 |
| ECUGP | ugpE | SFVMAFSITLGKITVSMLSAF* | 80 | 100 |
| ECUGP | ugpE | FPLRNLFFWMIFITLMLPVEV* | 107 | 127 |
| ECUGP | ugpE | DSYAGLTLPLMASATATFLFR* | 143 | 163 |
| ECUGP | ugpE | TNLAALFVITFIYGWNQYLWP* | 198 | 218 |
| ECUGP | ugpE | SVMVALLTLIPPVVIVLVMQR* | 249 | 269 |
| ECWITAG | kgtP gene | FYVYSFCSLYFAHIFFPSGNT* | 38 | 58 |
| ECWITAG | kgtP gene | QTAGVFAAGFLMRPIGGWLFG* | 64 | 84 |
| ECWITAG | kgtP gene | SMLLSVCMMCFGSLVIACLPG* | 95 | 115 |
| ECWITAG | kgtP gene | FQYVTLIGGQLLALLVVVVLQ* | 164 | 184 |
| ECWITAG | kgtP gene | WGWRIPFALGAVLAVVALWLR* | 195 | 215 |
| ECWITAG | kgtP gene | AFIMVLGFTAGSLCFYTFTTY* | 244 | 264 |
| ECWITAG | kgtP gene | ASGIMTAALFVFMLIQPLIGA* | 280 | 300 |
| ECWITAG | kgtP gene | SMLCFGSLAAIFTVPILSALQ* | 310 | 330 |
| ECWITAG | kgtP gene | AAFGLVMCALLIVSFYTSISG* | 337 | 357 |
| ECWITAG | kgtP gene | ALGVGLSYAVANAIFGGSAEY* | 370 | 390 |
| ECWITAG | kgtP gene | AFFWYVTLMAVVAFLVSLMLH* | 403 | 423 |
| HPCOPA | copA | LALAVIFTLFVVYLSMGAMLS* | 84 | 104 |
| HPCOPA | copA | HSNFLNACLQLIGTLIVMHWG* | 118 | 138 |
| HPCOPA | copA | NMSSLIAIGTSAALISSLWQL* | 155 | 175 |
| HPCOPA | copA | SYGHYYFESVCVILMFVMVGK* | 188 | 208 |
| HPCOPA | copA | VPSVIAIAILAFVVWLIIAPK* | 348 | 368 |
| HPCOPA | copA | FGIALEVFVSVLVISCPSCFR* | 375 | 395 |
| HPCOPA | copA | FWAFCYNSVFIPLACGVLYKA* | 688 | 708 |
| HPCOPA | copA | LSPAIAGLAMSLSSVSVVLNS* | 712 | 732 |
| IN | f1geneI | RITLPFVGTLYSLITGSKMPL* | 151 | 171 |
| IN | f1geneI | RLCLAIGFASAFTYSYITQPK* | 253 | 273 |
| MLSRA | sra | AGMVPLLTVLGNIIHTMALKA* | 103 | 123 |
| MLSRA | sra | NSISSSTASSIMPIVASQVTE* | 279 | 299 |
| MLSRA | sra | TAHSVPAGCSAITTAVSGPLE* | 358 | 378 |
| NPCBAABC | cbaD | LALIAIYFLLIAGLWIFTYFV* | 20 | 40 |
| NPCBAABC | cbaB | LWLAAAMVLIVGFIATITYGS* | 9 | 29 |
| NPCBAABC | cbaA | LAFFTSFAALAVGAALGLIQV* | 17 | 37 |
| NPCBAABC | cbaA | GVLLVITFTIFFLVGIFTWAV* | 60 | 80 |
| NPCBAABC | cbaA | WYGLMTLGTVLAAIPMVGGLI* | 97 | 117 |
| NPCBAABC | cbaA | AHPLFYLGLAVFVVGTWLAGV* | 136 | 156 |
| NPCBAABC | cbaA | PLPTFMVLTTMIFWYLSSIGV* | 175 | 195 |
| NPCBAABC | cbaA | GHAVVYFWLMPAYMMWYIMLP* | 227 | 247 |
| NPCBAABC | cbaA | LARVVFVLFLVLSTPTGIHHQ* | 258 | 278 |
| NPCBAABC | cbaA | NTMFLLLPSLLTAFTVVASME* | 295 | 315 |
| NPCBAABC | cbaA | FTGMALAGLMFAAAAFSGMVN* | 340 | 360 |
| NPCBAABC | cbaA | HLTVGTAVALTFMAVSYWFLP* | 380 | 400 |
| NPCBAABC | cbaA | VALAQVVLWFVGMTFMSNAMH* | 412 | 432 |
| NPCBAABC | cbaA | GILLFVSTLLFVLVVVMTVLG* | 471 | 491 |
| NPCBAABC | cbaA | TAIAVILVILAYALPLASIIS* | 524 | 544 |
| NPCBAABC | cbaC | VSRAGLLVAIAFLVPVIVELR* | 3 | 23 |
| NPCBAABC | cbaC | LQTVLLALFAVAALLAWAVAP* | 36 | 56 |
| PDCOX1 | cox2Pd | GVAAVMSLGVATMTAVPALAQ* | 10 | 30 |
| PDCOX1 | cox2Pd | VLYIITAVTIFVCLLLLICIV* | 67 | 87 |
| PDCOX1 | cox2Pd | VIWTLVPVLILVAIGAFSLPI* | 108 | 128 |
| PPHLYX | hlyD | LVAYFIMGFLVIAFILSVLGQ* | 59 | 79 |
| PRARO | arsB | MLLAGAIFILTIVLVIWQPKG* | 1 | 21 |
| PRARO | arsB | IGWSATLGAVLALASGVIHIA* | 24 | 44 |
| PRARO | arsB | NIVWNATATFIAVIIISLLLD* | 51 | 71 |
| PRARO | arsB | GRGRLLFTYIVLLGAAVAALF* | 89 | 109 |
| PRARO | arsB | DGAALILTPIVIAMLLALGFS* | 112 | 132 |
| PRARO | arsB | TTLAFVMAAGFISDTASLPLI* | 135 | 155 |
| PRARO | arsB | VMVPVDIAAIIATLVMLHLFF* | 178 | 198 |
| PRARO | arsB | TGWVVLLLLLVGFFVLEPMGI* | 226 | 246 |
| PRARO | arsB | APWQIVIFSLGMYLVIYGLRN* | 277 | 297 |
| PRARO | arsB | LWAATLGTGFLTALLSSIMNN* | 316 | 336 |
| PRARO | arsB | PKITPIGSLATLLWLHVLSQK* | 372 | 392 |
| PRARO | arsB | RTGIVMTLPVLFVTLAALALR* | 403 | 423 |
| RCFDXC | ORFU2 | DFLLVLLSTALVNNVVLVKFL* | 3 | 23 |
| RCFDXC | ORFU2 | AAIGMGLATTFVITVASAACW* | 37 | 57 |
| RCFDXC | ORFU2 | LRILSMILVIAAIVQFIETVM* | 72 | 92 |
| RCFDXC | ORFU2 | GIYLPLITTNCAVLGLPLMYI* | 104 | 124 |
| RCFDXC | ORFU2 | TLSGFGASVGFTLVLVIFAGM* | 134 | 154 |
| RCFDXC | ORFU2 | PIAFVSAGLLGLAFMGFAGLV* | 171 | 191 |
| RLDCTBD | dctB | MWWTYAALAFLAVVASLWTSG* | 25 | 45 |
| RLDCTBD | dctB | RMLALLILLPLLAGAAFLLRR* | 319 | 339 |
| RMEXOHKL | exoH | TVLLPFLIWSGSFFVVVYAIQ* | 90 | 110 |
| RMEXOHKL | exoH | FLRDLMLCILLSPLLALLVSR* | 148 | 168 |
| RMEXOHKL | exoH | RVTLLALLAYAILPLPNGIFL* | 171 | 191 |
| RMEXOHKL | exoH | SILFGFSAGIYASLHGVNIKM* | 194 | 214 |
| RMEXOHKL | exoH | FAAPIAAGFLAIAVVIAVGLY* | 218 | 238 |
| RMEXOHKL | exoH | ALRTSIAGIIGSWAISELLVR* | 251 | 271 |
| RMEXOHKL | exoH | LSFWIFCGHYPLLVLFWMIWN* | 284 | 304 |
| RMEXOHKL | exoH | YPLFYFTAPFIAIAILVASHN* | 311 | 331 |
| RVPRCH | rceHRv | IAQLVWYAQWLVIWTVVLLYL* | 12 | 32 |
| RVPRCLM | rceLRv | YFVGFFGVSAIFFIFLGVSLI* | 30 | 50 |
| RVPRCLM | rceLRv | GWHVPLAFCVPIFMFCVLQVF* | 115 | 135 |
| RVPRCLM | rceLRv | MSSVSFLFVNAMALGLHGGLI* | 175 | 195 |
| RVPRCLM | rceLRv | LGLFLASNIFLTGAFGTIASG* | 233 | 253 |
| SABLA1 | blaR1 | AKLLIMSIVSFCFIFLLLLFF* | 2 | 22 |
| SABLA1 | blaR1 | VWYLTLLAGLIPFIPIKFSLF* | 38 | 58 |
| SABLA1 | blaR1 | IDNICTVIWIVLVIILSFKFL* | 102 | 122 |
| SABLA1 | blaR1 | DTLHLIIFNIFSIIMSYNPLV* | 210 | 230 |
| SABLA1 | blaR1 | KLILIFICIFTFLLMVIQSQF* | 307 | 327 |
| SGSCAA | p29k | ALITAIVIGVVAGAVGCFIIL* | 18 | 38 |
| SGSCAA | p29k | INFFIGAITFGLLASIIITYI* | 64 | 84 |
| SGSCAA | p29k | TAIGITFSSFLALGIILISVA* | 94 | 114 |
| SGSCAA | p29k | DMWISIGVGILVLLVISIFFK* | 135 | 155 |
| SGSCAA | p29k | MKVNFYHYLLMILLTLVSVTA* | 172 | 192 |
| SGSCAA | p29k | SVGTILIVAMLITPAATAYLY* | 195 | 215 |
| SGSCAA | p29k | TMILLSSALGAGASVLGLFIG* | 221 | 241 |
| SGSCAA | p29k | AGSSIVTSALIFLVSFFIAPK* | 248 | 268 |
| SGSCAA | scaA | CRFLVLLLLAFVGLAACSSQK* | 4 | 24 |
| SHSTPSMP | SmpB | KSTFIIPIIIMLLMVVQAIIS* | 16 | 36 |
| SHSTPSMP | SmpB | SAFSGFSWFIFLLIIQASTII* | 51 | 71 |
| SHSTPSMP | SmpB | FSLLYFIIVIVVGIILWAIFF* | 104 | 124 |
| SHSTPSMP | SmpB | LGTYVGTWLVLSITLLISCAM* | 148 | 168 |
| SHSTPSMP | SmpB | AVGIVLYFATSILSGILTIVV* | 176 | 196 |
| SHSTPSMP | SmpB | LFIGNIVYIIIFLILVVFVFK* | 232 | 252 |
| SPENDA | endA | RQTLIGLLVLLLLSTGSYYIK* | 6 | 26 |
| SPPBPX | pbpX | SLLSVFVFAIFLVNFAVIIGT* | 30 | 50 |
| STOADGAB | oadG | FTLMFLGMGFVLAFLFLLIFA* | 12 | 32 |
| STOADGAB | oadB | LGAGQAIMLLVSLLLLWLAIA* | 16 | 36 |
| STOADGAB | oadB | FEPLLLLPIGFGGLLSNIPEA* | 39 | 59 |
| STOADGAB | oadB | VAIGSGVAPLVIFMGVGAMTD* | 129 | 149 |
| STOADGAB | oadB | TLLLGAAAQFGIFATVLGALT* | 159 | 179 |
| STOADGAB | oadB | YFGLISFTLPQAAAIGIIGGA* | 182 | 202 |
| STOADGAB | oadB | LLGAIAVAAYSYMALVPLIQP* | 218 | 238 |
| STOADGAB | oadB | ILFPVVLLMLVALLLPDAAPL* | 265 | 285 |
| STOADGAB | oadB | GLINIVTIFLGLSVGAKLVAD* | 311 | 331 |
| STOADGAB | oadB | LGILLLGVIAFGIGTAAGVLM* | 339 | 359 |
| STOADGAB | oadB | MGPNVAGVIGSAIAAGVMLKY* | 408 | 428 |
| STOPPAF | oppC | AVASLIVLFLIALFVTVAPML* | 39 | 59 |
| STOPPAF | oppC | ISLMVGIAAALVAVIVGTLYG* | 103 | 123 |
| STOPPAF | oppC | ILNSFPFMFFVILLVTFFGQN* | 143 | 163 |
| STOPPAF | oppC | NVLGVVVVYASLLVPSMILFE* | 217 | 237 |
| STOPPAF | oppC | WLLLFPAGFLVVTLFCFNFIG* | 269 | 289 |
| STPGTP | pgtP | LSATQIGLLSSCMLIAYGISK* | 61 | 81 |
| STPGTP | pgtP | VGLGFSSAFWIFAALVVFNGL* | 109 | 129 |
| STPGTP | pgtP | NVGGGIVAPIVGAAFAILGSE* | 162 | 182 |
| STPGTP | pgtP | ASYIVPACVAVIFALIVLVLG* | 187 | 207 |
| STPGTP | pgtP | FVYMVRFGMISWLPIYLLTVK* | 266 | 286 |
| STPGTP | pgtP | SVAFLFFEWAAIPSTLLAGWL* | 294 | 314 |
| STPGTP | pgtP | MPLAMICMALIFVCLIGYWKS* | 324 | 344 |
| STPGTP | pgtP | LLMVTIFAAIVGCLIYVPQFL* | 347 | 367 |
| STPGTP | pgtP | GLRGFMSYIFGASLGTSLFC"* | 386 | 406 |
| VCTOXR | toxrVc | RLFILIAVLLPLAVLLLTNPS* | 182 | 202 |
